# Cost-effectiveness analysis of sugemalimab combined with chemotherapy as first-line treatment for advanced gastric cancer

**DOI:** 10.3389/fpubh.2025.1620663

**Published:** 2025-07-31

**Authors:** Lian Tang, LongXun Zhu, ShaoQing Zhan, Yong Chen, Pan-Feng Feng

**Affiliations:** ^1^Department of Pharmacy, Affiliated Hospital 2 of Nantong University, and First People's Hospital of Nantong City, Nantong, China; ^2^Nantong Key Laboratory of Innovative Research on Rheumatology and Immunology, Nantong, China; ^3^Nantong Clinical Medical College of Kangda College of Nanjing Medical University, Nantong, China

**Keywords:** sugemalimab, first-line treatment, advanced gastric cancer, partitioned survival model, cost-effectiveness analysis

## Abstract

**Objective:**

Based on findings from the GEMSTONE-303 trial, the sugemalimab plus capecitabine and oxaliplatin regimen showed superior clinical efficacy compared to chemotherapy alone in advanced gastric cancer patients. This economic evaluation study assesses the cost-effectiveness of sugemalimab combination therapy within China’s healthcare system framework.

**Methods:**

A partitioned survival model was constructed based on data from the GEMSTONE-303 study, with a cycle length of 3 weeks. The model simulated patients’ direct medical costs and quality-adjusted life years (QALYs) over a 10-year period. The incremental cost-effectiveness ratio (ICER) was used as the evaluation metric, comparing the ICER against the willingness-to-pay (WTP) threshold (3 times China’s per capita GDP in 2024, 287,391 CNY/QALY). One-way sensitivity analysis and probabilistic sensitivity analysis were conducted to assess the robustness of the results.

**Results:**

The base-case analysis showed that the sugemalimab regimen provided greater health benefits compared to the placebo group (1.36 QALYs vs. 1.24 QALYs) but incurred significantly higher costs (271,041.24 CNY vs. 44,174.69 CNY), yielding an ICER of 1,890,554.58 CNY/QALY. One-way sensitivity analysis indicated that the utility values for the progressive disease (PD) state, progression-free survival (PFS) state, and the cost of sugemalimab had the most substantial impact on the ICER. Probabilistic sensitivity analysis demonstrated stable results, with a 0% probability that the sugemalimab combination regimen was cost-effective.

**Conclusion:**

Under the current economic conditions in China, sugemalimab combined with chemotherapy as a first-line treatment for advanced gastric cancer is not cost-effective.

## 1 Introduction

Gastric cancer remains a prevalent malignant tumor worldwide with relatively poor prognosis, posing a serious threat to human health (1). According to statistics from the International Agency for Research on Cancer (IARC), there were approximately 968,000 new gastric cancer cases and 660,000 deaths globally in 2022, with both incidence and mortality ranking fifth among all cancers (2). Over 70% of new gastric cancer cases occur in Asia, with about 50% concentrated in Eastern Asia, predominantly in China (3). China accounts for 37.0% of global gastric cancer cases and 39.4% of related deaths. The established first-line regimen for unresectable advanced gastric cancer and GEJ malignancies [which account for over 70% of total gastric cancer cases in China (4)] consisted of fluoropyrimidine-based chemotherapy combined with platinum agents, yielding suboptimal survival outcomes with overall survival (OS) of only 1 year (5, 6). Novel developments in immune checkpoint blockade therapy, with particular focus on PD-1/PD-L1 pathway inhibition, have yielded encouraging therapeutic outcomes (7–9).

Sugemalimab is a fully human IgG4 (s228p) monoclonal antibody that specifically binds PD-L1 while preserving FcγRI engagement. This unique design facilitates macrophage-mediated antibody-dependent cellular phagocytosis (ADCP) through cross-linking of PD-L1 + tumor cells with FcγRI-expressing effector cells (10). GEMSTONE 303 was a Phase 3, randomized, double-blind, placebo-controlled study conducted across 54 clinical trial sites in China (11). This study evaluated the safety and efficacy of sugemalimab combined with capecitabine and oxaliplatin (CAPOX) compared to placebo plus CAPOX in patients with unresectable locally advanced or metastatic gastric or gastroesophageal junction adenocarcinoma whose programmed death-ligand 1 (PD-L1) combined positive score (CPS) was 5 or higher. The newly released results demonstrated that, compared to placebo plus CAPOX, sugemalimab combined with CAPOX significantly improved both median progression-free survival [15.6 months vs. 12.6 months, hazard ratio (HR) = 0.75, 95% confidence interval (CI) (0.61, 0.92)] and median overall survival [7.6 months vs. 6.1 months, HR = 0.66, 95% CI (0.54, 0.81)]. Additionally, the incidence of grade ≥3 treatment-related adverse events was similar between the two groups (53.9% vs. 50.6%), with manageable safety, indicating significant clinical benefits of this treatment regimen.

Given that the price of sugemalimab may impose a significant economic burden on gastric cancer patients and China’s healthcare system, it is necessary to evaluate its cost-effectiveness under the current pricing. Therefore, based on the GEMSTONE 303 trial, this study employs a three-state partitioned survival model from the perspective of China’s healthcare system to explore its reasonable pricing in alignment with its clinical value, aiming to provide a reference for future national medical insurance negotiations.

## 2 Materials and methods

### 2.1 Target population and treatment regimen

The characteristics of the target population in this study were consistent with those of the phase III randomized controlled trial GEMSTONE-303 (11). Eligible patients were aged 18–75 years with histologically confirmed unresectable locally advanced or metastatic gastric/gastroesophageal junction adenocarcinoma. Patients were required to provide fresh or archived tumor samples for PD-L1 assessment, have a baseline PD-L1 combined positive score (CPS) ≥ 5, and measurable or evaluable disease per Response Evaluation Criteria in Solid Tumors version 1.1 (RECIST v1.1), with at least one measurable lesion. Exclusion criteria included known HER2-positive status, disease progression within 6 months after prior systemic therapy, or adjuvant/neoadjuvant chemotherapy.

The treatment regimen followed the GEMSTONE-303 study. Patients were randomly assigned to receive either sugemalimab or placebo (1,200 mg via intravenous infusion every 21 days for up to 24 months). Additionally, oxaliplatin (130 mg/m^2^, intravenous infusion) was administered on day 1 of each cycle, and capecitabine (1,000 mg/m^2^, orally twice daily) was given on days 1–14 of each cycle for six cycles. Treatment continued until disease progression, unacceptable toxicity, or withdrawal of consent. Since subsequent treatment options were not disclosed in the study, paclitaxel was selected as the second-line therapy for all patients based on the National Comprehensive Cancer Network (NCCN) guidelines, Chinese Society of Clinical Oncology (CSCO) guidelines, and relevant literature (12–14).

### 2.2 Model structure

**Figure 1:**
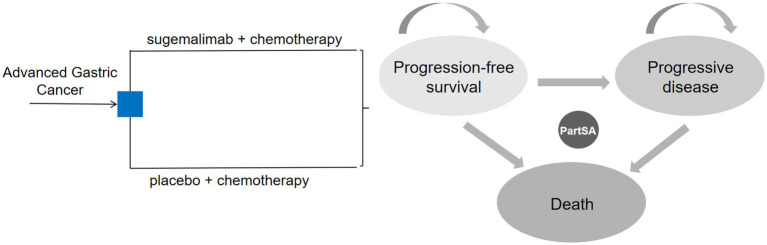
Partition survival model. PartSA, partitioned survival approach.

We constructed the partitioned survival model using TreeAge Pro software (2022 version), with reference to relevant published studies (15, 16), comprising three health states: progression-free survival (PFS), progressive disease (PD), and death. Transitions between states were assumed to be irreversible. All patients entered the model in the PFS state. Upon transitioning to PD, patients discontinued the current treatment and switched to a predefined subsequent therapy. The model cycle terminated when patients entered the death state. The state transition diagram is shown in Figure 1.

In alignment with the GEMSTONE-303 dosing schedule, the model cycle length was set at 3 weeks. Given the poor prognosis of advanced metastatic gastric cancer, with a 5-year survival rate of only 5% (17), the model time horizon was set at 10 years. Following the recommendations of the China Guidelines for Pharmacoeconomic Evaluations (2020) (18), an annual discount rate of 5% was applied for costs and utilities. The willingness-to-pay (WTP) threshold was defined as three times the 2024 per capita gross domestic product (GDP) in China (287,391 CNY). The primary model outputs included total costs, quality-adjusted life years (QALYs), and the incremental cost-effectiveness ratio (ICER). The economic value of the treatment was assessed by comparing the ICER against the WTP threshold.

### 2.3 Survival analysis

**Figure 2:**
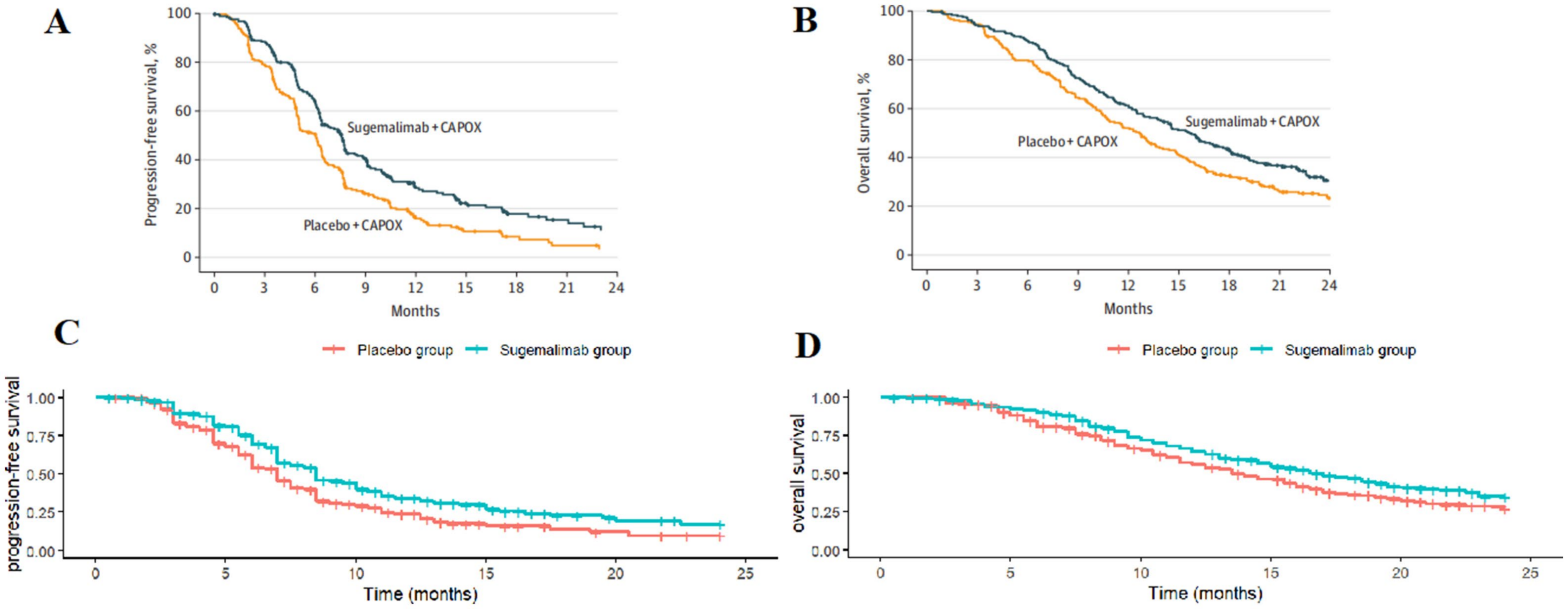
Optimal curve fitting extrapolation of two treatment schemes. **(A)** Original PFS curve; **(B)** Original OS curve; **(C)** Simulated PFS curve; **(D)** Simulated OS curve.

Patient survival data were extracted from the GEMSTONE-303 study. The WebPlotDigitizer 4.7 tool was used to digitize data points from the original survival curves, and individual patient-level data were reconstructed using R software (version 4.4.1). These data were then fitted to survival models to extrapolate survival outcomes beyond the clinical follow-up period (19, 20). Various parametric distributions (Exponential, Gompertz, Weibull, Log-logistic, and Lognormal) were tested to fit the reconstructed patient-level data. The optimal distribution was selected based on the Akaike Information Criterion (AIC), Bayesian Information Criterion (BIC), and visual inspection. The estimated overall survival (OS) and progression-free survival (PFS) curves for both treatment groups are shown in Figure 2, and the fitted distribution parameters are presented in Tables 1, 2. The Lognormal distribution was ultimately chosen to fit the PFS and OS curves for both the sugemalimab plus chemotherapy and placebo plus chemotherapy groups.

**Table 1:** AIC and BIC of survival curve in two groups.

Survival curve	Model criteria	Exponential	Gompertz	Weibull	Log-logistic	Lognormal
Treatment groupPFS curve	AIC	1018.929	1010.703	982.798	956.833	955.359
	BIC	1022.414	1017.673	989.767	963.802	962.329
Control groupPFS curve	AIC	987.556	975.180	940.240	909.163	905.087
	BIC	991.029	982.125	947.184	916.108	912.031
Treatment groupOS curve	AIC	1164.337	1146.220	1130.099	1122.948	1122.906
	BIC	1167.822	1153.189	1137.068	1129.918	1129.876
Control groupOS curve	AIC	1208.737	1194.474	1174.506	1162.169	1157.596
	BIC	1212.209	1201.418	1181.450	1169.114	1164.541

**Table 2:** Parameter distribution of survival curve in two groups.

Survival curve	Optimal fitting distribution	Mean	SD
Treatment group PFS curve	Lognormal	2.231524	0.757559
Control group PFS curve	Lognormal	1.964002	0.685329
Treatment group OS curve	Lognormal	2.841857	0.851456
Control group OS curve	Lognormal	2.638810	0.817169

### 2.4 Costs and utilities

This study adopted the perspective of the Chinese healthcare system, considering only direct medical costs, including drug costs, follow-up costs (laboratory tests, imaging examinations), best supportive care (BSC), end-of-life care, and the costs of managing adverse drug reactions (ADRs) with an incidence of ≥5% and grade ≥3 (as reported in the GEMSTONE-303 study). The detailed cost items are listed in Table 3. Drug prices were based on the median 2025 tender prices from the Yaozhi database. The costs of the two treatment regimens were calculated according to the dosing schedules used in the trial. For weight- or body surface area (BSA)-based dosing, we assumed a patient weight of 59 kg (21) and a BSA of 1.72 m^2^ (22). Since the GEMSTONE-303 trial did not report health utility values for Chinese gastric cancer patients, utility parameters were derived from published literature, with PFS and PD state utilities set at 0.797 and 0.577, respectively (23). The costs of managing ADRs (24) were calculated by multiplying the incidence rates by the unit cost per adverse event.

**Table 3:** Model parameters.

Variable	Baseline Value	Minimum	Maximum	Distribution	Reference
Cost (CNY)					
Sugemalimab/mg	20.625	16.500	24.750	Gamma	https://www.yaozh.com/
Oxaliplatin/mg	2.087	1.670	2.504	Gamma	https://www.yaozh.com/
Capecitabine/g	6.083	4.866	7.300	Gamma	https://www.yaozh.com/
Laboratory and imaging test	231.00	184.80	277.20	Gamma	(23)
Terminal care	1469.00	1175.20	1762.80	Gamma	(23)
BSC	248.00	198.40	297.60	Gamma	(23)
Decreased platelet count	1505.92	1204.74	1807.10	Gamma	(24)
Decreased neutrophil count	115.01	92.00	138.01	Gamma	(23)
Decreased white blood cell count	467.86	374.29	561.43	Gamma	(24)
Anemia	468.19	374.55	561.82	Gamma	(24)
Incidence rate of adverse reaction/%					
Decreased platelet count (Treatment group)	18.30	14.64	21.96	Beta	(11)
Decreased neutrophil count (Treatment group)	14.10	11.28	16.92	Beta	(11)
Anemia (Treatment group)	10.80	8.64	12.96	Beta	(11)
Decreased white blood cell count (Treatment group)	6.60	5.28	7.92	Beta	(11)
Decreased platelet count (Control group)	16.00	12.8	19.2	Beta	(11)
Decreased neutrophil count (Control group)	14.30	11.44	17.16	Beta	(11)
Anemia (Control group)	7.20	5.76	8.64	Beta	(11)
PFS	0.797	0.638	0.956	Beta	(24)
PD	0.577	0.462	0.692	Beta	(23)
Discount rate/%	5	0	8	Beta	(18)
Weight	59.00	47.20	70.80	Normal	(21)
Body surface area/m^2^	1.72	1.38	2.06	Normal	(22)

### 2.5 Sensitivity analysis

To assess the robustness of the model results, one-way sensitivity analysis (OWSA) and probabilistic sensitivity analysis (PSA) were conducted. In the OWSA, parameters were varied by ±20% from their baseline values to evaluate their impact on model outcomes, with results presented in a tornado diagram. For the PSA, 1,000 Monte Carlo simulations were performed, assuming Gamma distributions for cost parameters and Beta distributions for adverse event rates and utility values. The results were presented as cost-effectiveness scatter plots and cost-effectiveness acceptability curves.

## 3 Results

### 3.1 Base-case analysis

The 10-year model results showed that, compared with the placebo plus chemotherapy regimen, the sugemalimab plus chemotherapy regimen provided an incremental effectiveness of 0.12 QALYs at an incremental cost of ¥226,866.55, resulting in an ICER of ¥1,890,554.58 per QALY. This value exceeded the predefined WTP threshold (¥287,391), indicating that the sugemalimab plus chemotherapy regimen was not cost-effective as a first-line treatment for advanced gastric cancer. The results are presented in Table 4.

**Table 4:** Baseline results.

Parameters	Treatment group	Control group
Total cost/CNY	271041.24	44174.69
Incremental cost/CNY	226866.55	
Effect/QALYs	1.36	1.24
Incremental effect/QALYs	0.12	
ICER, CNY/QALY	1890554.58	

### 3.2 Scenario analysis

Scenario analyses with varying time horizons demonstrated that while the ICER for sugemalimab progressively decreased with extended timeframes, all values remained above 3 times China’s 2024 per-capita GDP (Table 5). Sensitivity analyses employing alternative survival distributions for PFS and OS curves consistently yielded ICERs exceeding the predefined WTP threshold across all scenarios (Table 6).

**Table 5:** ‌Results of scenario analyses under different simulation time horizons.

Simulation time horizons	Group	Total cost/CNY	Incremental cost/CNY	Effect/QALYs	Incremental effect/QALYs	ICER, CNY/QALY
3 years	Treatment group	262395.51	229049.56	0.95	0.07	3272136.571
	Control group	33345.95		0.88		
4 years	Treatment group	264693.22	228469.4	1.06	0.08	2855867.5
	Control group	36223.82		0.98		
5 years	Treatment group	266395.09	228039.69	1.14	0.09	2533774.333
	Control group	38355.40		1.05		
6 years	Treatment group	267721.05	227704.88	1.20	0.1	2277048.8
	Control group	40016.17		1.10		
7 years	Treatment group	268789.30	227435.16	1.25	0.1	2274351.6
	Control group	41354.14		1.15		
8 years	Treatment group	269670.65	227212.62	1.30	0.12	1893438.5
	Control group	42458.03		1.18		
9 years	Treatment group	270410.87	227025.71	1.33	0.12	1891880.917
	Control group	43385.16		1.21		
10 years	Treatment group	271041.24	226866.55	1.36	0.12	1890554.583
	Control group	44174.69		1.24		
15 years	Treatment group	273154.60	226332.94	1.46	0.13	1741022.615
	Control group	46821.66		1.33		

**Table 6:** Effects of different simulation distribution approaches on the ICER.

Distributions	Incremental cost/CNY	Incremental effect/QALYs	ICER, CNY/QALY
Exponential	230548.17	0.14	1646772.64
Gompertz	230106.3	0.06	3835105
Weibull	229682.7	0.04	5742067.5
Log-logistic	229380.35	0.11	2085275.91
Lognormal	226866.55	0.12	1890554.58

### 3.3 One-way sensitivity analysis

**Figure 3:**
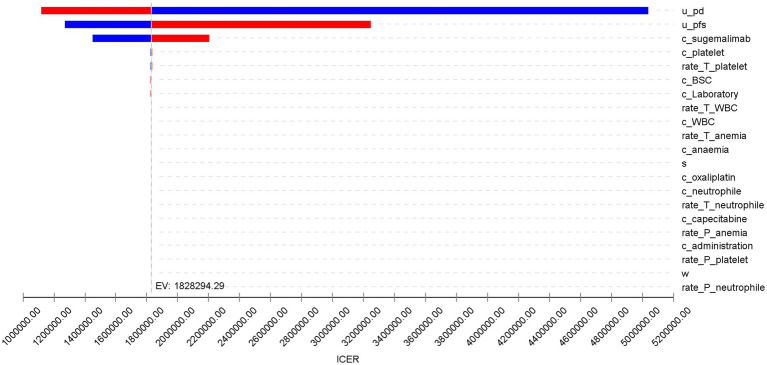
Tornado diagram for one-way sensitivity analysis.

The results of the one-way sensitivity analysis are shown in Figure 3. The utility values of the PD state, PFS state, and the cost of sugemalimab had a significant impact on the model outcomes, while other variables had minimal influence on the ICER. However, regardless of the variations in the predefined parameter ranges, the sugemalimab plus chemotherapy regimen consistently lacked cost-effectiveness as a first-line treatment for advanced gastric cancer, suggesting the robustness of the base-case analysis results.

### 3.4 Probabilistic sensitivity analysis

**Figure 4:**
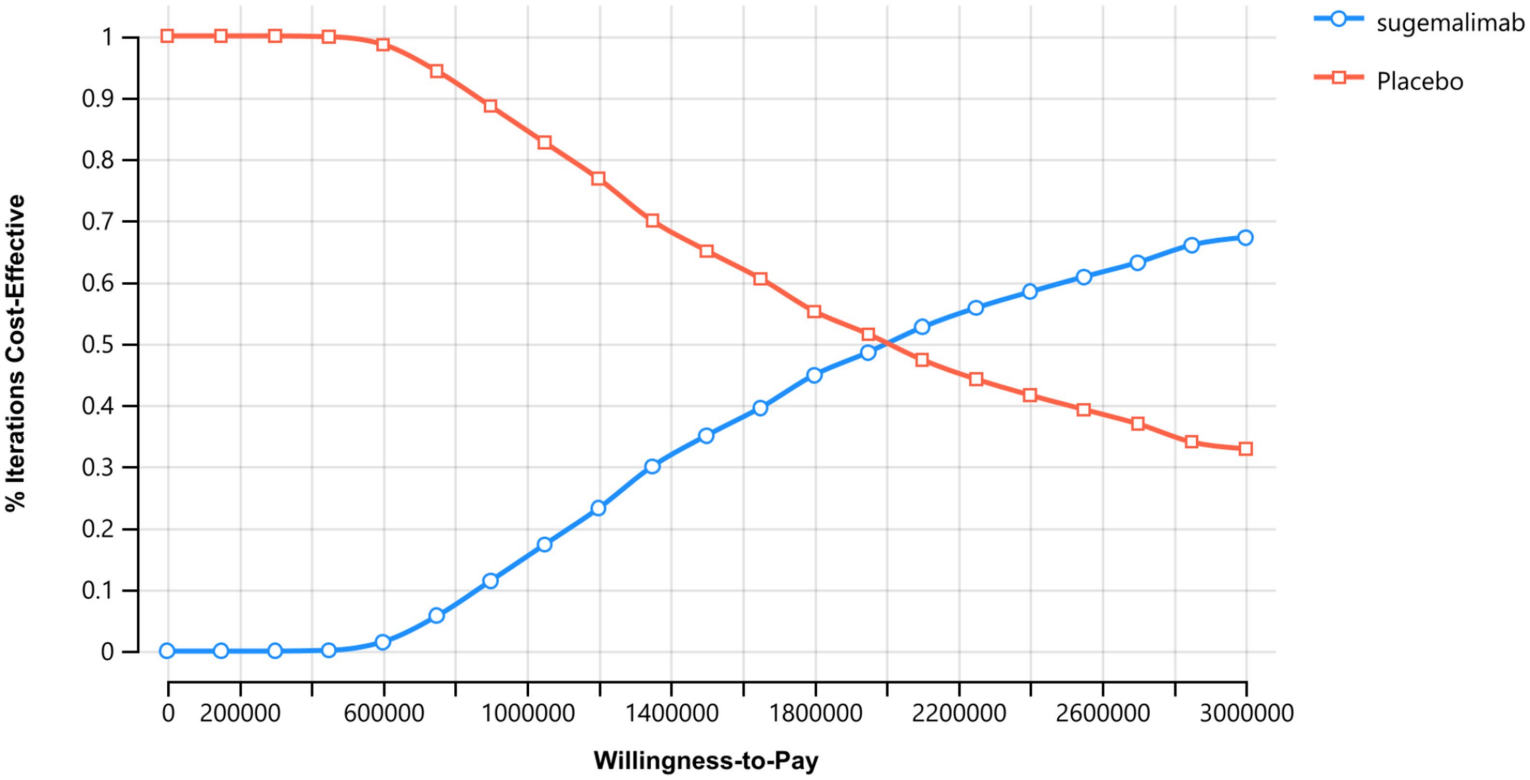
Acceptability curves.

**Figure 5:**
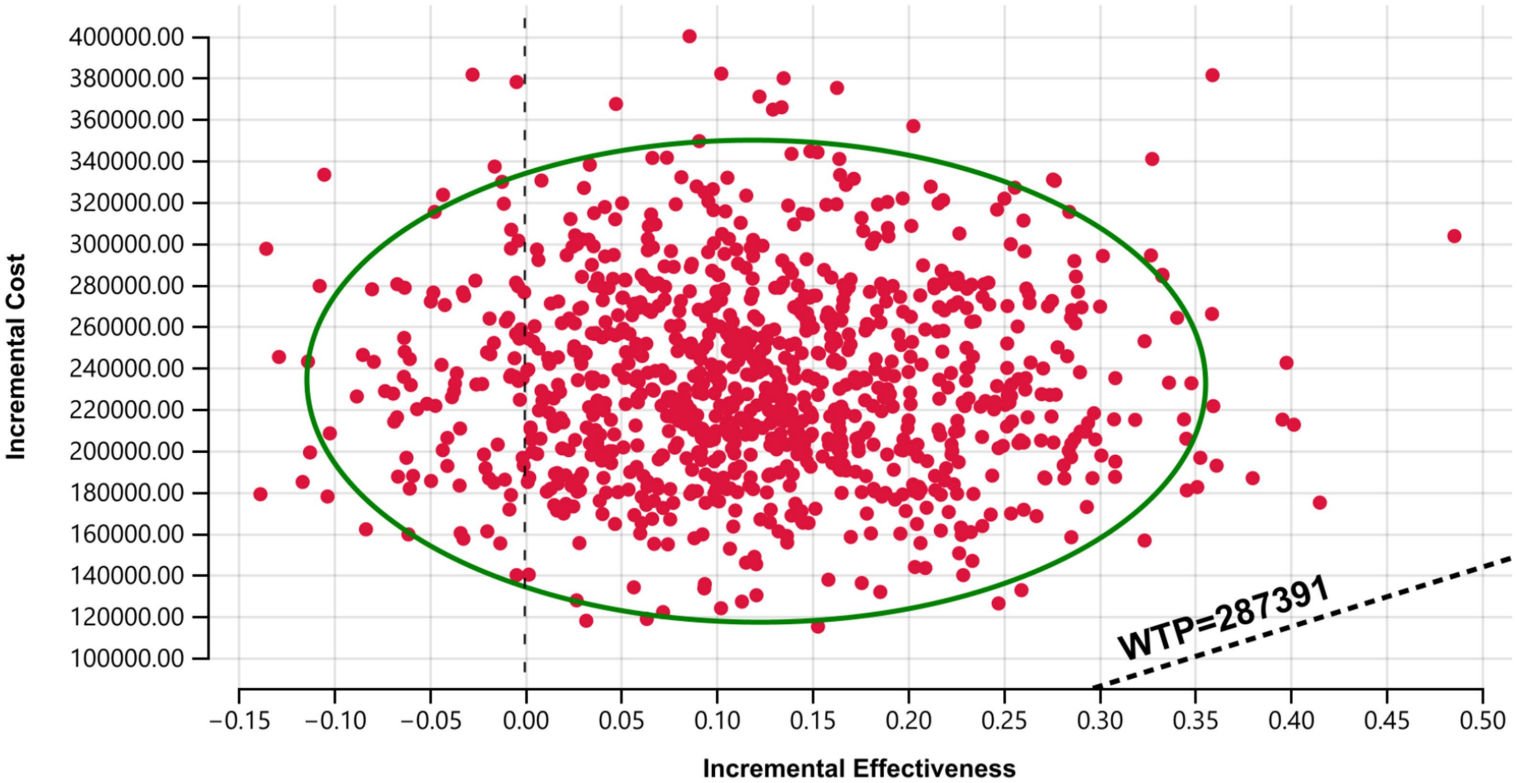
Cost-effective scatter plot. Results of Monte Carlo probabilistic sensitivity analysis showing incremental cost-effectiveness of sugemalimab + chemotherapy versus placebo + chemotherapy.

The probabilistic sensitivity analysis results are presented in Figures 4, 5. The cost-effectiveness acceptability curve revealed that the economic value of sugemalimab plus chemotherapy as a first-line treatment for advanced gastric cancer increased with higher WTP thresholds, whereas the economic value of the placebo plus chemotherapy regimen declined. When the WTP was below ¥420,000, the probability of sugemalimab plus chemotherapy being cost-effective was 0%. When the WTP increased to approximately ¥2,000,000, the probabilities of cost-effectiveness for both regimens became equal. The cost-effectiveness scatter plot demonstrated that all incremental cost-effectiveness points fell above the three-times GDP per capita line in China (2024), further confirming that the sugemalimab plus chemotherapy regimen lacked economic advantage.

## 4 Discussion

The present study evaluated the cost-effectiveness of sugemalimab combined with chemotherapy as a first-line treatment for advanced gastric cancer in China, utilizing a partitioned survival model based on data from the GEMSTONE-303 trial. The results demonstrated that, despite providing incremental clinical benefits (0.12 QALYs), the regimen’s high incremental cost (¥226,866.55) resulted in an ICER of ¥1,890,554.58 per QALY, far exceeding China’s WTP threshold (¥287,391). Although the GEMSTONE-303 trial demonstrated significant improvements in PFS and OS, these benefits primarily resulted from prolonged disease stabilization rather than a substantial enhancement in quality of life, which may have reduced the QALY weighting. Furthermore, long-term survivors receiving immunotherapy may experience immune-related adverse events, potentially diminishing utility values. Consequently, the ICER was considerably higher than the predefined WTP threshold. This finding suggests that, under current pricing, sugemalimab is not a cost-effective option for advanced gastric cancer treatment within China’s healthcare system.

The robustness of these conclusions was confirmed through sensitivity analyses. One-way sensitivity analysis identified the utility values of PD and PFS states, as well as the cost of sugemalimab, as the most influential parameters on the ICER. However, even under extreme variations of these parameters, the regimen remained economically unviable. Probabilistic sensitivity analysis further reinforced this outcome, showing a 0% probability of cost-effectiveness at WTP thresholds below ¥420,000. Only at an unrealistically high WTP (¥2,000,000) did the sugemalimab regimen achieve parity with chemotherapy alone, highlighting the need for substantial price reductions to align with China’s economic realities.

These findings have significant implications for healthcare policy and clinical practice. While the GEMSTONE-303 trial established the clinical efficacy of sugemalimab, its high cost poses a barrier to widespread adoption in resource-limited settings like China. Potential strategies to improve cost-effectiveness include price negotiations, patient assistance programs, or biomarker-driven approaches targeting subpopulations with higher PD-L1 expression (e.g., CPS ≥ 10), who may derive greater benefit. Such measures could enhance the regimen’s value proposition and facilitate its inclusion in national reimbursement schemes.

Our study has several notable strengths. First, to our knowledge, this is the first comprehensive cost-effectiveness analysis of sugemalimab combined with chemotherapy for advanced gastric cancer in the Chinese healthcare context, providing crucial evidence for policymakers and clinicians. Second, our model utilized robust clinical data from the GEMSTONE-303 trial, ensuring that the survival and efficacy inputs were derived from a high-quality randomized controlled trial. Additionally, we conducted extensive sensitivity analyses (both one-way and probabilistic), which confirmed the stability of our findings across a wide range of parameter uncertainties.

This study has the following limitations: (1) The PFS and OS curves were extrapolated by fitting parametric distributions. Although this approach can predict survival trends beyond the follow-up period of the GEMSTONE-303 trial, the extrapolated survival data rely on assumptions inherent to parametric models. Consequently, the actual survival benefits may differ from the model predictions. (2) The original study did not specify the exact second-line chemotherapy regimen. In this analysis, subsequent treatments were selected based on clinical guidelines, which may not fully reflect real-world prescribing practices. (3) Only grade ≥3 adverse reactions with an incidence >3% were included. While this may introduce discrepancies compared to actual clinical outcomes, the one-way sensitivity analysis demonstrated that the costs of managing adverse events had minimal impact on the results. Thus, this limitation is unlikely to alter the study’s conclusions. (4) Findings are specific to China’s healthcare pricing and reimbursement policies. The cost-effectiveness of sugemalimab may differ in countries with higher willingness-to-pay thresholds or alternative drug pricing structures. Despite these limitations, our study provides valuable insights into the economic viability of sugemalimab for advanced gastric cancer in China.

## 5 Conclusion

In conclusion, while sugemalimab represents a promising therapeutic advance for advanced gastric cancer, its current cost renders it economically unsustainable in China. Policymakers and manufacturers must collaborate to develop pricing strategies that balance innovation with affordability, ensuring patient access without overburdening the healthcare system. This study provides a critical foundation for such discussions and underscores the importance of economic evaluations in guiding healthcare decision-making.

## Data Availability

The original contributions presented in the study are included in the article/Supplementary material, further inquiries can be directed to the corresponding authors.
